# Clinical manifestation and treatment of small intestinal lymphangioma: A single center analysis of 15 cases

**DOI:** 10.3389/fmed.2022.975698

**Published:** 2022-09-23

**Authors:** Zhen-zhen Wang, Ling-yan Shen, Jing-jing Zhou, Jia-li Tang, Li-ping Ye, Chen-bo Shen, Shao-wei Li, Xian-bin Zhou

**Affiliations:** ^1^Department of Gastroenterology, Taizhou Hospital of Zhejiang Province Affiliated to Wenzhou Medical University, Linhai, Zhejiang, China; ^2^Key Laboratory of Minimally Invasive Techniques and Rapid Rehabilitation of Digestive System Tumor of Zhejiang Province, Taizhou Hospital Affiliated to Wenzhou Medical University, Linhai, Zhejiang, China; ^3^Institute of Digestive Disease, Taizhou Hospital of Zhejiang Province Affiliated to Wenzhou Medical University, Linhai, Zhejiang, China

**Keywords:** lymphangioma, small intestine, diagnosis, treatment, endoscope

## Abstract

**Background:**

Small intestinal lymphangioma is a very rare benign lesion. Thus far, the literature on small intestinal lymphangioma has mainly involved case reports. The present study retrospectively examined the clinical features of patients with a pathological diagnosis of small intestinal lymphangioma.

**Materials and methods:**

From January 2010 to January 2021, 15 patients were pathologically diagnosed with small intestinal lymphangioma. The age, gender, clinical manifestation, computed tomography (CT) findings, endoscopic findings, localization of the lesion, treatment method, complications, and follow-up were retrospectively analyzed.

**Results:**

Most of the patients had no symptoms, and those with symptoms had melena or abdominal pain. Lymphangioma was located in the duodenum in nine cases (60.0%), jejunum in two (13.3%), jejunal-ileal junction with mesentery involvement in one (6.7%) and ileum in three (20.0%). Three cases (20.0%) had multiple lesions, and the other 12 (80.0%) had single lesions. The median size of the lesions was 0.8 cm. Thirteen cases were found by endoscopy, and nine cases of them had white-colored spots on the surface. Ten cases (66.7%) underwent endoscopic treatment, three (20.0%) underwent surgical treatment, and two (13.3%) were followed up. Postoperative acute pancreatitis developed in one patient after endoscopic resection of duodenal papillary lymphangioma; postoperative abdominal bleeding occurred in one patient with jejunal lymphangioma who underwent partial small bowel resection.

**Conclusion:**

Small intestinal lymphangioma is extremely rare, and its clinical manifestations are non-specific. Endoscopy is of great value in the diagnosis of small intestinal lymphangioma. Depending on the clinical manifestations, the size, location and scope of the lesions, follow-up, endoscopic treatment and surgery can be selected.

## Introduction

Lymphangioma is a benign tumor of the lymphatic system characterized by the presence of dilated lymphatic spaces. It mostly occurs in the head, axillae and neck and also in the spleen, bone and other parenchymal organs, while rarely occurring in the gastrointestinal tract. Small intestinal lymphangioma is extremely rare, comprising less than 1% of all cases (1).

The etiology and pathogenesis of lymphangioma remain unclear. It is generally believed that it originates from the isolation of lymphoid tissue during the embryonic period and is not connected with the normal lymphoid system. Acquired factors, such as abdominal trauma, abdominal surgery, radiotherapy, inflammation and parasites, may also lead to lymphangioma (2). Histopathologically, lymphangiomas are classically categorized into three types: cavernous, capillary and cystic lymphangioma.

Small intestinal lymphangioma is a rare cause of abdominal pain and gastrointestinal hemorrhaging (1, 3, 4). However, the diagnosis of lymphangioma in the small intestinal is occasionally difficult preoperatively, especially in the jejunum and ileum, even if a biopsy is performed under endoscopy.

Various clinical manifestations, computed tomography (CT) findings and endoscopic findings of small intestinal lymphangioma have been described in case reports and a few small series. Furthermore, most studies on small intestinal lymphangioma thus far have been limited to cases requiring surgical or endoscopic resection, thereby indirectly excluding smaller incidental or endoscopically detected lesions.

To our knowledge, this study examined the largest set of patient data including smaller-sized small intestinal lymphangiomas that were incidentally detected. This study retrospectively examined the clinical features of patients with pathologically confirmed small intestinal lymphangioma.

## Materials and methods

This retrospective study was approved by the ethics committee of Taizhou Hospital of Zhejiang Province (Linhai, China). The study included 15 consecutive patients with pathologically confirmed small intestinal lymphangioma from January 2010 to January 2021 The data of 15 patients were retrospectively and electronically abstracted. The age, gender, clinical manifestation, computed tomography (CT) findings, endoscopic findings, localization of the lesion, treatment method, complications and follow-up were retrospectively analyzed.

Data were analyzed using the SPSS software program (version 20.0; SPSS Inc., Chicago, IL, USA). Descriptive statistics were used for this study. The median was used for variables with a skewed distribution, while the mean was used in cases with a normal distribution of variables. Numerated data are expressed as case numbers and percentages (%).

## Results

Patient demographics and clinical characteristics are summarized in Table 1. There were six males (40.0%) and nine females (60.0%) with an average age of 52.1 (range, 16–72) years old. Two patients (13.3%) presented with melena (including one whose symptoms were considered associated with other ileal lesions), three (20.0%) presented with abdominal pain (including one whose symptoms were caused by a gallstone and one with symptoms caused by Crohn’s disease), and the other 10 (66.7%) had no symptoms.

**TABLE 1:** Clinical characteristics of 15 small bowel lymphangioma cases.

Number	Sex	Age, years	Clinical presentation	Maximum diameter of lesion, cm	Site	Number of lesions	Therapy	Complication	Hospital stay, days	Follow-up period, months
1	F	56	No symptoms	0.5	Descending duodenum	1	Endoscopic resection	No	1	6
2	F	49	No symptoms	0.8	Terminal ileum	2	Endoscopic resection	No	3	10
3	F	36	No symptoms	0.5	Descending duodenum	1	Endoscopic resection	No	7	24
4	M	59	No symptoms	0.5	Descending duodenum	2	Endoscopic resection	No	6	33
5	M	55	Melena	6	Jejunum	1	Surgery	Postoperative abdominal bleeding	25	38
6	M	56	No symptoms	0.4	Descending duodenum near papilla	1	Endoscopic resection	No	6	44
7	M	59	No symptoms	0.6	Descending duodenum	1	Endoscopic resection	No	7	49
8	F	51	No symptoms	1.0	Descending duodenum near papilla	1	Endoscopic resection	No	4	52
9	F	70	Abdominal pain, nausea (caused by Crohn’s disease)	6.0	Upper ileum	1	Follow-up	No	22	52
10	F	61	No symptoms	1.5	Terminal ileum	1	Endoscopic resection	No	4	56
11	F	51	No symptoms	0.6	Descending duodenum	1	Endoscopic resection	No	4	65
12	M	45	No symptoms	1.4	Duodenal papilla	1	Endoscopic resection	Acute pancreatitis	23	77
13	M	16	Abdominal pain, abdominal distension, and anal discharge	12.0	Jejuno ileal junction and mesentery	1	Surgery	No	22	92
14	F	45	Abdominal pain (caused by gallstone)	0.5	Jejunum	1	Surgery	No	23	106
15	F	72	Melena (caused by other ileal lesions)	0.8	Duodenal bulb and descending duodenum	Multiple	Follow-up	No	6	115

Lymphangioma was located in the duodenum in nine cases (60.0%), jejunum in two cases (13.3%), jejunal-ileal junction with mesentery involvement in 1 case (6.7%) and ileum in three cases (20.0%). Three cases (20.0%) had multiple lesions, and the other 12 (80.0%) had single lesions. The median lesion size was 0.8 (range, 0.4–12) cm.

All patients were negative for immunologic series and tumor markers (AFP, CEA, CA199, and CA125). Patients with gastrointestinal hemorrhaging showed fecal occult blood positivity and had anemia, with a minimum hemoglobin level of 39 g/l. Seven cases underwent abdominal CT. One showed intestinal volvulus and intestinal obstruction. There were no positive findings in the remaining cases.

**FIGURE 1:**
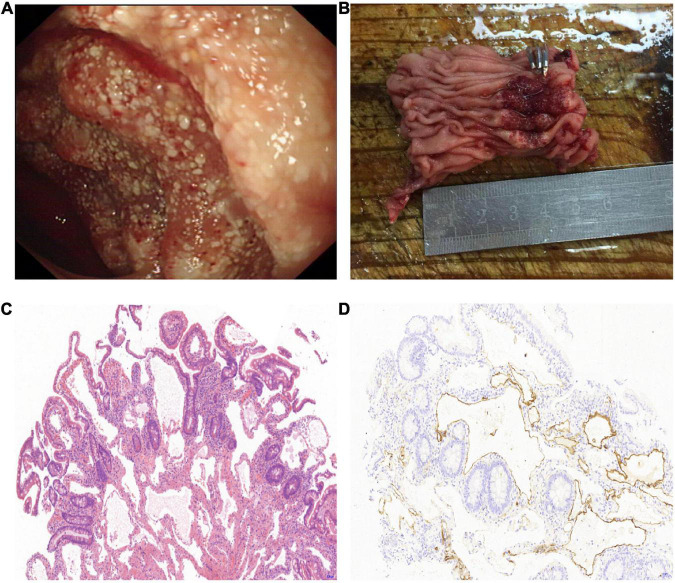
Surgically resected lymphangioma of jejunum due to gastrointestinal bleeding. **(A)** An irregular protuberant lesion of 5.0 cm × 6.0 cm in the upper segment of the ileum, with white-colored spots on the surface. **(B)** Photograph of segmental ileal resection, including the lymphangioma lesions. **(C)** Morphology showing that the lesions were composed of variably sized cysts in the mucosal lamina propria and submucosa, and the lumen was filled with lymphatic fluid (hematoxylin–eosin staining, 10× magnification). **(D)** D2-40 immunostaining shows positive reactivity for the endothelial cells lining the lymphatic spaces in the muscular layer (10× magnification).

Thirteen cases of lymphangioma were detected by endoscopy, of which nine were found by gastroscopy (one was also found by capsule endoscopy); two cases of terminal ileum lymphangioma were found by colonoscopy; and two cases of jejunum and upper ileum lymphangioma were found by capsule endoscopy. Macroscopically, all 13 cases of small intestinal lymphangioma showed protrusion. White-colored spots on the surface were seen in nine cases (Figure 1), while the remaining four showed a smooth surface.

Endoscopic ultrasonography was performed in six patients whose lesions were in the descending duodenum. One case originated from the muscularis and submucosa, and the other five originated from the submucosa. Four cases had uneven echo, and two cases had uniform echo. Four cases had mixed echo, and two were hypoechoic. All six cases had clear boundaries.

Endoscopic resection was performed in 10 patients (66.7%), including endoscopic submucosal dissection (ESD) in three cases, high-frequency electric resection in five cases, a forceps biopsy in one case, and snare cauterization in one case. Three cases (20.0%) underwent surgical resection. The other two cases (13.3%) were followed up, as the symptoms were associated with multiple small intestinal ulcers and Crohn’s disease (*n* = 1 each).

One patient developed postoperative acute pancreatitis after endoscopic resection of duodenal papillary lymphangioma, and the condition improved after fasting, acid inhibition and inhibition of pancreatic enzyme secretion. Postoperative abdominal bleeding occurred in one patient with jejunal lymphangioma who underwent partial small intestinal resection, and laparotomy and hemostasis were performed as treatment. All 15 patients recovered and were discharged.

The median hospital stay was six (range, 1–25) days. The median follow-up period after the procedure was 52 months (range, 6–115 months). No residual or recurrent lesions were detected during the follow-up period in any of the 13 patients who underwent endoscopic or surgical resection. The small intestinal lymphangioma lesions showed no obvious changes in the remaining two patients who were followed up.

## Discussion

Lymphangiomas usually occur in the head, neck and axilla as well as in the parenchymal organs, such as the liver, spleen and bone but rarely in the digestive tract, especially in the small intestine, which accounts for less than 1% of all cases (1). However, with the increasing popularity of endoscopy, more and more small intestinal lymphangiomas may be detected. Indeed, in this study, 10 cases were asymptomatic and were incidentally detected by gastroscopy or colonoscopy.

Small intestinal lymphangioma usually has no clinical symptoms when it is small. The clinical presentation of small intestinal lymphangioma is unspecific and often involves acute or chronic hemorrhaging. The pathogenesis of spontaneous hemorrhaging of lymphangioma is still unknown. One possible reason is that lymphangioma consists of blood vessels and lymphatic channels of various sizes (1). Intestinal obstruction, volvulus and intussusception can occur when the lymphangioma grows in a circular pattern along the bowel or involves the mesentery. Other symptoms of small intestinal lymphangioma are infection, perforation, rupture, and protein-losing gastroenteropathy (5–7). In the present study, there were 10 asymptomatic cases with relatively small lesions (maximum diameter ≤ 1.5 cm) that were incidentally detected by endoscopy. The lesions of the two cases with lymphangioma-related symptoms were large, with maximum diameters of 6.0 cm in one and 12.0 cm in the other. One case was located at the jejunal-ileal junction, involving the mesentery, with a maximum diameter of 12.0 cm and showing volvulus and intestinal obstruction. The other case was located in the jejunum, with a maximum diameter of 6.0 cm, and presented with repeated gastrointestinal hemorrhaging.

Patients with small intestinal lymphangioma usually presented with no or non-specific symptoms. Thus, relying solely on a clinical manifestation may lead to a missed diagnosis or misdiagnosis. Imaging examinations may help detect and localize lesions. The CT appearances of lymphangiomas in the gastrointestinal tract are a well-demarcated oval mass of low attenuation beneath the submucous membrane. Cysts between the enhanced mucous membrane and serous membrane were homogeneous, watery, and non-enhancing, showing a stratifying effect. Some gastrointestinal lymphangiomas may manifest as submucosal pedunculated proliferative lesions (8). In the present study, seven cases underwent abdominal CT. However, only one showed intestinal volvulus and intestinal obstruction. This may have been due to it being a small lesion, with no filling of the intestinal cavity or contraction of the intestinal wall.

Endoscopy is valuable in the diagnosis of small intestinal lymphangioma. The typical endoscopic findings show a soft submucosal mass with white-colored spots on the surface (9). Most of our series had the above typical endoscopic manifestations. Iwamuro et al. (10) performed magnifying endoscopy to observe duodenal lymphangioma, and elongated microvessels were seen on the surface of the white spots. Exudation of milky fluid after a biopsy of lymphangioma was also observed in theirs and others’ reports (10–12). Consequently, the endoscopic features may be the consequence of the duodenal villi being dilated and the microvessels being stretched due to the retention of chyle (10).

Lymphangioma in the duodenal bulb and descending part is easy to detect by gastroscopy. All nine cases of duodenal lymphangioma were detected by gastroscopy in our study. However, it is difficult to detect such lesions when they are located far from the descending duodenum. The application of capsule endoscopy and enteroscopy has significantly improved the diagnostic rate of small intestinal lymphangioma. Capsule endoscopy helps detect the lesion location, which plays an important role in further enteroscopy or surgical exploration. In the present study, capsule endoscopy was performed in three cases, and lymphangioma was detected in all three cases, two of which were pathologically diagnosed by a biopsy under enteroscopy.

Endoscopic ultrasonography (EUS) is helpful in the diagnosis and differential diagnosis of small intestinal lymphangioma. The characteristic view of lymphangioma on EUS is a cystic tumor originating from the third layer and is an anechoic–hypoechoic tumor with septum (13). However, Hizawa et al. (14) reported that in one patient with dilated lymphatic channels of < 1 mm, EUS revealed a homogeneous echogenic mass within the second and third layers. One of our series had similar endosonographic manifestations that originated from the second and third layers. Furthermore, the internal echo patterns of lymphangiomas vary depending on the size of the dilated lymphatic channels (14).

Because small intestinal lymphangioma is benign, surveillance is a safe option if the lesion is small or asymptomatic. However, Lymphangiomas are submucosal lesions that sometimes the pathology is only known after resection (15). Also, some patients are worried about the risk of growing lesions and ask for treatment. When the lesions cause complications, further treatment is required. Most cases are treated surgically. Complete surgical resection is the main form of treatment whenever possible, while the surrounding normal tissues are sometimes removed. When there is local invasion, complete resection is sometimes impossible (3). Lymphangiomas smaller than 2 cm that are accessible endoscopically and have no local invasion may be treated by endoscopic resection (16). In our study, 10 patients with lesions showing a maximum diameter of ≤ 1.5 cm were treated by endoscopy. One patient suffered postoperative acute pancreatitis after endoscopic resection of duodenal papillary lymphangioma, and their condition improved after conservative treatment. For duodenal papillary lymphangioma, there are risks of acute pancreatitis and biliary tract infection after the operation. Biliary stent and pancreatic stent placement after lesion resection may reduce the risks.

However, there are some limitations in this study. First, the sample size was small due to the rarity of the tumor. Second, this study was a single-center retrospective study.

## Conclusion

Small intestinal lymphangioma is extremely rare, and its clinical manifestations are non-specific. The possibility of small intestinal lymphangioma should be considered in patients with unexplained gastrointestinal hemorrhaging, intestinal obstruction, volvulus, and intussusception. Endoscopy is of great value in the diagnosis of small intestinal lymphangioma. Depending on the clinical manifestations, the size, location and scope of the lesions, follow-up, endoscopic treatment or operation can be selected.

## Data availability statement

The original contributions presented in this study are included in the article/supplementary material, further inquiries can be directed to the corresponding authors.

## Ethics statement

The study was reviewed and approved by the Ethics Committee of Taizhou Hospital of Zhejiang Province affiliated to Wenzhou Medical University Institutional Review Board (approval No. K20210622). The patients/participants provided their written informed consent to participate in this study.

## Author contributions

Z-ZW, L-YS, and J-JZ participated in the clinical treatment. J-LT and S-WL wrote the original draft. L-PY, C-BS, and X-BZ undertook validation, writing, review, and editing. All authors contributed to the article and approved the submitted version.
